# Effects of GLP-1 receptor agonists on neurological complications of diabetes

**DOI:** 10.1007/s11154-023-09807-3

**Published:** 2023-05-26

**Authors:** Natalia García-Casares, Guillermo González-González, Carlos de la Cruz-Cosme, Francisco J Garzón-Maldonado, Carmen de Rojas-Leal, María J Ariza, Manuel Narváez, Miguel Ángel Barbancho, Juan Antonio García-Arnés, Francisco J. Tinahones

**Affiliations:** 1 Facultad de Medicina, Departamento de Medicina, Universidad de Málaga, Málaga, Spain; 2 Instituto de Investigación Biomédica de Málaga (I.B.I.M.A), Málaga, Spain; 3 Centro de Investigaciones Médico-Sanitarias (C.I.M.ES), Málaga, Spain; 4 Hospital Universitario Virgen de la Victoria de Málaga, Málaga, Spain; 5 Departamento de Medicina, Facultad de Medicina, Universidad de Málaga, Centro de Investigaciones Médico Sanitarias (C.I.M.E.S), Universidad de Málaga, Instituto de Investigación Biomédica de Málaga (IBIMA), Campus Universitario de Teatinos s/n., Málaga, 29010 España; 6 Departamento de Medicina, Facultad de Medicina, Universidad de Málaga, Instituto de Investigación Biomédica de Málaga (IBIMA), Campus Universitario de Teatinos s/n., Málaga, 29010 España

**Keywords:** Cognitive impairment, Stroke, Cardiovascular disease, Peripheral neuropathy, Cognitive impairment, Alzheimer’s disease

## Abstract

Emerging evidence suggests that treatment with glucagon-like peptide-1 receptor agonists (GLP-1 RAs) could be an interesting treatment strategy to reduce neurological complications such as stroke, cognitive impairment, and peripheral neuropathy. We performed a systematic review to examine the evidence concerning the effects of GLP-1 RAs on neurological complications of diabetes. The databases used were Pubmed, Scopus and Cochrane. We selected clinical trials which analysed the effect of GLP-1 RAs on stroke, cognitive impairment, and peripheral neuropathy. We found a total of 19 studies: 8 studies include stroke or major cardiovascular events, 7 involve cognitive impairment and 4 include peripheral neuropathy. Semaglutide subcutaneous and dulaglutide reduced stroke cases. Liraglutide, albiglutide, oral semaglutide and efpeglenatide, were not shown to reduce the number of strokes but did reduce major cardiovascular events. Exenatide, dulaglutide and liraglutide improved general cognition but no significant effect on diabetic peripheral neuropathy has been reported with GLP-1 RAs. GLP-1 RAs are promising drugs that seem to be useful in the reduction of some neurological complications of diabetes. However, more studies are needed.

## Introduction

People with diabetes have a high neurological risk, such as stroke, cognitive impairment, or diabetic neuropathy. In recent years we have witnessed the vertiginous progress in relation to new treatments for diabetes, which are not just limited to good glycaemic control, but can have beneficial effects on other organs such as the brain.

Glucagon-like peptide-1 receptor agonists (GLP-1 RAs) currently play an important therapeutic role in the treatment of type 2 diabetes. They present similar properties to the human peptide as they reduce blood glucose and glycosylated haemoglobin levels, favouring insulin receptor sensitivity and increasing insulin secretion. On the other hand, they can slow down gastric emptying, achieving an increase in satiety and a reduction in body weight. All the GLP-1-RAs (lixisenatide, liraglutide, semaglutide, exenatide, dulaglutide, albiglutide, efpeglenatide) are administered via subcutaneous (sc.) injection, except for semaglutide with both routes of administration, sc. and oral. Recent studies have shown that GLP-1 RAs also act on the nervous system, providing neuroprotective effects, mainly by controlling vascular risk factors and cardiovascular improvement [1].

The cardiovascular safety of GLP-1-RAs in patients with type 2 diabetes has been demonstrated in multiple randomized controlled trials [2, 3]. The US Food and Drug Administration and the European Medicines Agency have required safety studies of these drugs on cardiovascular risk in patients with diabetes, which have allowed us to know their cardiovascular effects, including stroke, and further our understanding of its benefits in patients with type 2 diabetes and high vascular risk.

On the other hand, cognitive impairment is one more complication in persons with diabetes, presenting a cognitive profile with alterations mainly in the speed of information processing, verbal and visual memory, attention, and executive function [4]. Neuroimaging studies with MRI and brain PET have shown correlations with these neuropsychological alterations and brain structural-functional alterations [5, 6]. Some studies suggest that there is an overlap between the pathophysiological mechanisms of Alzheimer’s disease and diabetes mediated by insulin resistance [7]. It is known that patients with Alzheimer’s disease present a decrease in insulin levels and an alteration of the signal in insulin receptors in the brain [7], favouring the deposition of amyloid beta and tau protein [8]. Experimental studies have shown the expression of GLP-1 receptors in areas such as the cerebral hippocampus, especially in the dendrites of the pyramidal cells of the CA1 and CA3 region of this structure, which is crucial in the cognitive processes of learning and memory [9, 10]. Other studies have demonstrated the neuroprotective role of GLP-1 analogues as they produce a reduction in oxidative stress and apoptosis, showing new brain connections and neuroplasticity [11].

Another complication of diabetes is peripheral neuropathy affecting the peripheral nervous system. It can affect about 50% of patients with diabetes [12]. Symptoms of peripheral neuropathy present with varying degrees of numbness, tingling, or pain primarily in the distal parts of the extremities [13]. An early assessment of peripheral polyneuropathy symptoms helps prevent ulcers, local infection, or sepsis, and even death [12]. Many risk factors for diabetic neuropathy have been identified, including the presence of cardiovascular risk factors [14] and studies have aimed to determine the possible benefit of GLP-1 RAs in the prevention or improvement of peripheral neuropathy.

This study aimed to perform a systematic review to identify the evidence relating the effects of GLP-1 RAs on neurological complications of diabetes (stroke, cognitive impairment, and peripheral neuropathy).

## Methods

### Search strategy

To undertake this systematic review, an exhaustive search was carried out through the Medline, Cochrane, and Scopus databases to identify those clinical trials related to the topic until January 2022. The keywords used were the same in the different databases: “GLP-1 Ras” [MeSH Terms] AND “stroke” [MeSH Terms] OR “cardiovascular disease” [MeSH Terms] OR “peripheral neuropathy” [MeSH Terms] OR “cognitive impairment” [MeSH Terms] OR “Alzheimer’s disease” [MeSH Terms].

Selection criteria.

**Fig. 1:**
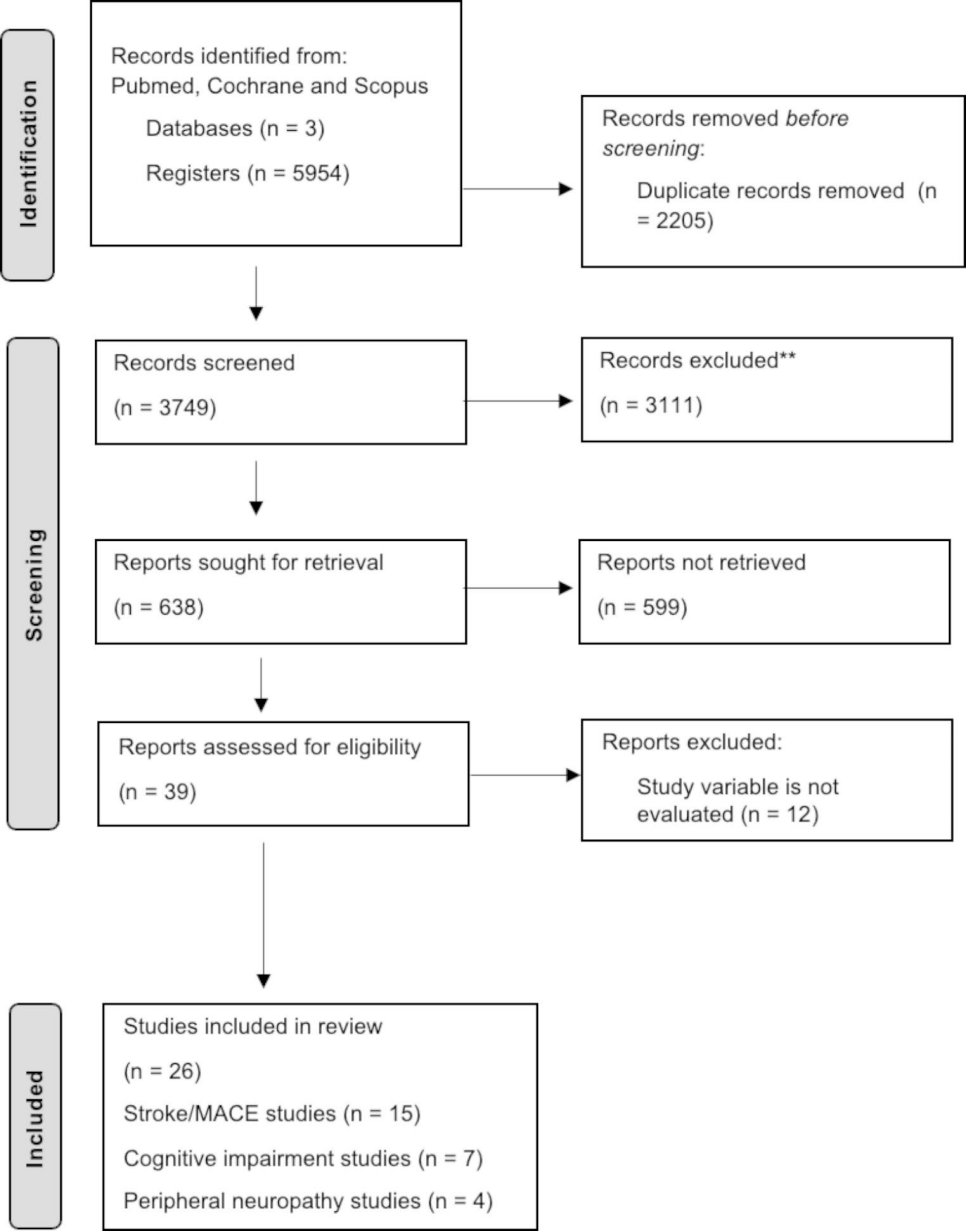
Flowchart of the strategy search

Studies were included in this systematic review if they met the following criteria: study design was a clinical trial or controlled clinical trial; participants were adults with a diagnosis of either type 1 or 2 diabetes mellitus with GLP-1 RA treatment and a history of stroke or cardiovascular events, cognitive impairment, or peripheral neuropathy. Each study had to detail the number of physical events and mental points and then compare the results with the other intervention group. It was also necessary to specify if either stroke or a major cardiovascular event (MACE) was measured in the cardiovascular field, and what kind of tests and imaging were used in both cognitive impairment and peripheral neuropathy, so the starting point of the participants was also required. The exclusion criteria for this systematic review were clinical cases, reviews, meta-analyses, letters to the editor, single case studies, communications to conferences, other neurological diseases that were not addressed in the systematic review, animal studies and articles not written in English or Spanish. This systematic review is reported according to the Preferred Reported Items for Systematic reviews and Meta-Analyses (PRISMA) [15]. The PRISMA diagram illustrates the selection process of the studies and shows reasons for exclusion (Fig. 1).

## Results

The search resulted in a total of 5954 articles, though after applying the inclusion and exclusion criteria, only 26 studies were finally selected.

### GLP-1 RAs and stroke/MACE

This systematic review included 8 studies related to GLP-1 RAs and stroke/MACE [16–23]. All were double blind, randomized, placebo-controlled trials, and all enrolled middle- or old-aged participants with a previous diagnosis of type 2 diabetes as well as cardiovascular risk factors or cardiovascular disease. The study measure was specifically stroke in almost all articles [16–26], except for the AMPLITUDE-0 trial [23] where it was MACE. Post-hoc analyses of these studies were included in the discussion (see Table 1).

**Table 1:** Glucagon-like peptide-1 receptor agonists and Stroke Mace/Events.

Study [Ref.]	Study design	Patients, n	Type of participants	Intervention	Study measure	Main findings	Conclusions
Pfeffer M.A. et al. [16] (2015)	Multicentre, randomized, double-blind, placebo-controlled trial; ELIXA	N = 6,068	Participants aged > 30 years with diagnosis of type 2 diabetes, who also had an acute coronary event within 180 days before screening.	Lixisenatide sc. (n = 3,034) vs. placebo (n = 3,034)	STROKE	-Lixisenatide sc. did not significantly reduce the number of stroke cases compared to placebo (p = 0.54).	Lixisenatide sc. could not be suitable for the reduction of MACE events nor for fatal or non-fatal stroke cases.
Marso S. P. et al. [19] (2016)	Randomized, double-blind, placebo-controlled trial; LEADER	N = 9,340	Participants aged > 50 years with diagnosis of type 2 diabetes and at least one cardiovascular condition or aged > 60 years with diagnosis of type 2 diabetes and at least one cardiovascular risk factor.	Once-daily liraglutide sc. (n = 4,668) or placebo (n = 4,672)	STROKE	-Liraglutide sc. did not significantly reduce the number of fatal stroke cases compared to placebo (p = 0.16). - Liraglutide sc. did not significantly reduce the number of non-fatal stroke cases compared to placebo (p = 0.3).	Liraglutide sc. could be suitable for the reduction of MACE events, but not for fatal or non-fatal stroke cases.
Verma S. et al. [57] (2019)	Post-hoc analysis of double-blind, randomized, placebo-controlled LEADER trial.	N = 9,340	Participants aged > 50 years with diagnosis of type 2 diabetes and at least one cardiovascular condition or aged > 60 years with diagnosis of type 2 diabetes and at least one cardiovascular risk factor.	Once-daily liraglutide sc. (n = 4,668) or placebo (n = 4,672)	MACE	-Liraglutide sc. was associated with a 15.7% relative risk reduction in total MACE and a 13.4% reduction in total expanded MACE compared with placebo.	Liraglutide sc. is more effective reducing expanded-MACE than MACE events in participants with type 2 diabetes and high risk of cardiovascular events.
Marso S. P. [54] (2020)	Post-hoc analysis of double-blind, randomized, placebo-controlled LEADER trial.	N = 9,340	Participants aged > 50 years with diagnosis of type 2 diabetes and at least one cardiovascular condition or aged > 60 years with diagnosis of type 2 diabetes and at least one cardiovascular risk factor.	Once-daily liraglutide sc. (n = 4,668) or placebo (n = 4,672)	STROKE	-No significant reduction in the number of strokes between liraglutide sc. and placebo in patients with no heart failure -No significant reduction in the number of non-fatal stroke cases was observed between liraglutide sc. and placebo in patients with NYHA functional class I-III	Liraglutide sc. reduces the number of MACE in patients with type 2 diabetes despite previous levels of NYHA functional class I to III HF, but not the number of strokes.
Buse J.B. et al. [58] (2020)	Post-hoc analysis of double-blind, randomized, placebo-controlled LEADER trial.	N = 9,340	Participants aged > 50 years with diagnosis of type 2 diabetes and at least one cardiovascular condition or aged > 60 years with diagnosis of type 2 diabetes and at least one cardiovascular risk factor.	Once-daily liraglutide sc. (n = 4,668) or placebo (n = 4,672)	MACE	-HbA1C changed as time-dependent covariate. - HbA1C updated mean as time-dependent covariate. -UACR changed as time-dependent covariate. -UACR updated mean as time-dependent covariate.	HbA1C, and to a lesser extent UACR reduction, are thought to be mediators of the MACE-preventive effects of liraglutide.
Marso S.P. et al. [20] (2016)	Randomized, double-blind, placebo-controlled trial. SUSTAIN-6.	N = 3,297	Participants aged > 50 years with type 2 diabetes with pre-existing cardiovascular disease, chronic heart failure or chronic kidney disease (stage 3 or higher), or aged > 60 years with diagnosis of diabetes and at least one cardiovascular risk factor.	Once-weekly semaglutide sc. (n = 1,648) versus placebo (n = 1,649).	STROKE	-Semaglutide sc. reduced the number of non-fatal stroke cases compared to placebo (p = 0.04).	Semaglutide sc. could be suitable for the reduction of MACE events and non-fatal stroke cases.
Leiter L. A. et al. [22] (2020)	Post-hoc analysis of randomized, double-blind, placebo-controlled LEADER and SUSTAIN 6 trials.	-LEADER TRIAL: N = 9,340 -SUSTAIN-6 TRIAL: N = 3,297	LEADER: Participants aged > 50 years with diagnosis of type 2 diabetes and at least one cardiovascular condition or aged > 60 years with diagnosis of type 2 diabetes and at least one cardiovascular risk factor. SUSTAIN-6: Participants aged > 50 years with type 2 diabetes with pre-existing cardiovascular disease, chronic heart failure or chronic kidney disease (stage 3 or higher), or aged > 60 years with diagnosis of diabetes and at least one cardiovascular risk factor.	Once-daily liraglutide sc. (n = 4,668) or placebo (n = 4,672) in LEADER trial. Once-weekly semaglutide sc. (n = 1,648) versus placebo (n = 1,649) in SUSTAIN 6 trial.	MACE	Primary MACE in LEADER: -BP normal (HR: 1.00; 95% CI, 0.75–1.32). -BP elevated (HR: 1.21; 95% CI, 0.87–1.68). -BP stage 1 hypertension (HR: 0.73; 95% CI, 0.60–0.90). -BP stage 2 hypertension (HR: 0.84; 95% CI, 0.72–0.99). Primary MACE in SUSTAIN-6: -BP normal (HR: 0.79; 95% CI, 0.40–1.56). -BP elevated (HR: 0.43; 95% CI, 0.20–0.95). -BP stage 1 hypertension (HR: 0.62; 95% CI, 0.37–1.03). -BP stage 2 hypertension (HR: 0.85; 95% CI, 0.60–1.21).	Liraglutide sc. and semaglutide sc. may be beneficial for patients with type 2 diabetes, irrespective of their baseline BP.
Verma S. et al. [21] (2020)	Post-hoc analysis of randomized, double-blind, placebo-controlled LEADER and SUSTAIN 6 trials.	-LEADER TRIAL: N = 9,340 -SUSTAIN-6 TRIAL: N = 3,297	LEADER: Participants aged > 50 years with diagnosis of type 2 diabetes and at least one cardiovascular condition or aged > 60 years with diagnosis of type 2 diabetes and at least one cardiovascular risk factor. SUSTAIN-6: Participants aged > 50 years with type 2 diabetes with pre-existing cardiovascular disease, chronic heart failure or chronic kidney disease (stage 3 or higher), or aged > 60 years with diagnosis of diabetes and at least one cardiovascular risk factor.	Once-daily liraglutide sc. (n = 4,668) or placebo (n = 4,672) in LEADER trial. Once-weekly semaglutide sc. (n = 1,648) versus placebo (n = 1,649) in SUSTAIN 6 trial.	MACE	-Participants with microvascular disease had an increased risk of major adverse cardiovascular events compared with those without microvascular disease in LEADER: (p = 0.0136) and in SUSTAIN 6: (p = 0.0064).	Microvascular disease was associated with an increased risk of MACE. Liraglutide sc. and semaglutide sc. reduce major CV outcomes in patients despite microvascular disease presence.
Holman R. R. et al. [17] (2017)	Pragmatic, event-driven, randomized, double-blind, placebo-controlled trial. EXSCEL	N = 14,752	Participants with diagnosis of type 2 diabetes and the trial was designed to have 70% of participants with previous cardiovascular events.	Exenatide sc. (n = 7,356) vs. placebo (n = 7,396).	STROKE	-Exenatide sc. did not significantly reduce the number of stroke cases (fatal or non-fatal) compared to placebo.	Exenatide sc. could not be suitable for reducing the number MACE events or stroke (fatal or non-fatal) cases.
Hernández A.F. et al. [18] (2018)	Double-blind, randomized, placebo-controlled trial. HARMONY	N = 10,793	Participants aged > 40 years and diagnosis of type 2 diabetes and cardiovascular disease.	Albiglutide sc. (n = 4,731) vs. placebo (n = 4,732).	STROKE	-Albiglutide sc. reduced the number of MACE (p = 0.0006). -Subgroup analysis showed that albiglutide sc. does not reduce the incidence of strokes.	Albiglutide sc. reduced the number of MACE but might not be suitable for reducing fatal or non-fatal stroke cases.
Gerstein H. et al. [21] (2019)	Multicentre, randomized, double-blind, placebo-controlled trial. REWIND	N = 9,901	Participants aged > 50 years with diagnosis of type 2 diabetes and either previous cardiovascular events or cardiovascular risk factors.	Dulaglutide sc. (n = 4,949) vs. placebo (n = 4,952).	STROKE	-Fatal stroke cases: the reduction in dulaglutide sc. group was bigger, but non-significant, than in placebo group (p = 0.34). -Non-fatal stroke cases: the reduction in dulaglutide sc. group was significantly bigger than in placebo group (p = 0.017).	Dulaglutide sc. may be suitable for reducing the number of non-fatal stroke cases.
Gerstein H.C. et al. [59] (2020)	Post-hoc analysis of the multicentre, double-blind, randomized, placebo-controlled REWIND trial.	N = 12,133	Participants aged ≥ 50 years with diagnosis of type 2 diabetes.	Dulaglutide sc. (n = 4,949) vs. placebo (n = 4,952).	STROKE	-Dulaglutide sc. reduced ischaemic stroke compared to placebo (p = 0·012). -Dulaglutide sc. reduced disabling-stroke compared to placebo (p = 0·042). -Dulaglutide sc. had no effect on haemorrhagic stroke compared to placebo (p = 0·89).	Long-term dulaglutide sc. use might reduce clinically relevant ischaemic stroke in people with type 2 diabetes but does not affect stroke severity.
Dagenais G.R. et al. [60] (2020)	Post-hoc analysis of the multicentre, double-blind, randomized, placebo-controlled REWIND trial.	N = 12,133	Participants aged ≥ 50 years with diagnosis of type 2 diabetes.	Dulaglutide sc. (n = 4,949) vs. placebo (n = 4,952).	MACE vs. Expanded MACE	- Dulaglutide sc. reduced the incidence of total MACE or non-cardiovascular deaths (p = 0.022). -Dulaglutide sc. reduced the incidence of total expanded MACE (p = 0.028).	Dulaglutide sc. reduced the event burden of CV or fatal outcomes in a population at moderate CV risk. The absolute reduction was greatest for the composite outcome that included an expanded definition of a MACE.
Husain M. et al. [22] (2019)	Event-driven, double-blind, randomized, placebo-controlled trial. PIONEER-6	N = 3,183	Participants aged > 50 years with diagnosis of type 2 diabetes and established cardiovascular disease or chronic kidney disease, or aged > 60 years with diagnosis of diabetes and cardiovascular risk factors.	Once-daily semaglutide oral (n = 1,591) or placebo (n = 1,592).	STROKE	-There were no significant differences between semaglutide oral or placebo in the number of strokes.	Stroke-preventive effect of semaglutide oral could not be proved.
Gerstein H. C. et al. [23] (2021)	Randomized, placebo-controlled trial. AMPLITUDE-0	N = 4,076	Participants aged ≥ 18 years old with diagnosis of type 2 diabetes and previous CV disease, or ≥ 50 years old with chronic kidney disease and one or more additional CV risk factors.	Efpeglenatide sc. (n = 2,717) vs. placebo (n = 1,359).	MACE	-There was a significant reduction in the number of MACE events in those participants treated with efpeglenatide sc. vs. placebo (p = 0.007).	Efpeglenatide sc. lowers the risk of MACE.

BP: Blood Pressure. CV: Cardiovascular. GLP-1 RAs: Glucagon-like Peptide-1 Receptor Agonists. HBA1C: Haemoglobin A1c. MACE: Major Adverse Cardiovascular Events. SC: subcutaneous

The primary objective of most of the large, double-blind, randomized, placebo-controlled trials that have evaluated the cardiovascular effects of GLP-1 RAs in patients with type 2 diabetes focused on evaluating the presence of a MACE, which was defined and generally included cardiovascular death, non-fatal myocardial infarction and nonfatal stroke. The subgroup analyses of each article found the following results in relation to the risk of stroke:

In the ELIXA lixisenatide trial that evaluated patients with acute coronary syndrome (n = 6068 patients with an acute coronary event within 180 days before screening), subcutaneous (sc.) lixisenatide had no effect on the risk of nonfatal stroke over a median 25-month follow-up [16].

In the EXSCEL cardiovascular event reduction study with exenatide, (n = 14,752 patients with or without established cardiovascular disease), extended-release exenatide also had no effect on the incidence of fatal or nonfatal stroke [17].

In the HARMONY study (n = 9463 patients with established coronary heart disease, cerebrovascular disease, or peripheral vascular disease), albiglutide, despite lowering MACE, had no effect on the risk of fatal or nonfatal stroke in the subgroup analysis during a follow-up of 1.6 years [18]. This drug was later withdrawn in 2018 due to commercial problems.

In the LEADER study with liraglutide, in the evaluation of cardiovascular events [n = 9340 patients, ≥ 50 years with established cardiovascular disease (coronary event, cerebrovascular event, or peripheral vascular disease), stage ≥ 3 chronic kidney disease, or chronic heart failure according to New York Heart Association class II or III or ≥60 years with at least one cardiovascular risk factor (CVRF)], liraglutide demonstrated a significant reduction in MACE by 13% at the expense of a reduction in cardiovascular mortality. However, subgroup analysis showed a non-significant trend in the effect on the incidence of fatal or nonfatal stroke or transient ischaemic attack during a follow-up of 3.8 years [19].

The SUSTAIN-6 study [20], which evaluated the long-term cardiovascular effects of semaglutide sc., in subjects with type 2 diabetes and HbA1c ≥ 7%, treated with up to two oral hypoglycaemic agents, with or without basal or premixed insulin, [n = 3297 patients, ≥ 50 years with established cardiovascular disease (coronary event, cerebrovascular event, or peripheral vascular disease), chronic kidney disease stage ≥ 3, or New York Heart Association class II or III chronic heart failure or ≥ 60 years with at least one CVRF)], found a significant reduction in the primary endpoint of MACE appearance by 26% (6.6% vs. 8.9%, HR 0.74, 95% CI 0.58–0.95). The subgroup analysis showed that this reduction of the primary objective was achieved at the expense of the reduction in nonfatal stroke, since treatment with semaglutide sc. reduced the risk of nonfatal stroke by 39% (1.6% vs. 2.7%, HR 0.61, 95% CI 0.38–0.99; p = 0.04) during a follow-up of 2.1 years. On the other hand, at the beginning of the study, 76.3% of the patients (n = 2514) were receiving preventive treatment for stroke: 85% of the patients were taking antiaggregants and 5.9% anticoagulants. Of the total of 3297 patients, during the study 71 patients presented a nonfatal stroke: 27 treated with semaglutide sc. [25 (1.5%) ischaemic and 2 (0.1%) haemorrhagic] vs. 44 with placebo [37 (2.2%) ischaemic, 4 (0.2%) haemorrhagic, and 3 (0.2%) unclassifiable stroke].

The REWIND study [21] with dulaglutide sc. [n = 9901 patients, ≥ 50 years with established cardiovascular disease or ≥ 60 years with at least two CVRFs], met the primary efficacy endpoint, showing that weekly dulaglutide significantly reduced MACE (12.0% vs. 13.4%, HR 0.88, 95% CI 0.79–0.99; p = 0.026), with a significant 24% reduction in nonfatal stroke (3.2% vs. 4.1%, HR 0.76, 95% CI 0.61–0.95; p = 0.017) during a follow-up of 5.4 years.

In the PIONEER-6 study with oral semaglutide [n = 3183 patients, ≥ 50 years with established cardiovascular or renal disease, or ≥ 60 years with CVRFs], no significant efficacy of oral semaglutide could be demonstrated in the reduction in nonfatal stroke (HR 0.74, 95% CI 0.35–1.57) during a 2.1-year follow-up [22].

Finally, in AMPLITUDE-0 with efpeglenatide [n = 4076 patients, ≥ 18 years with previous cardiovascular disease or ≥ 50 years with chronic kidney disease and 1 or more CVRFs], the efficacy objective was also achieved, demonstrating a significant decrease in MACE (7.0% vs. 9.2%, HR 0.73, 95% CI 0.58–0.92; p = 0.007). However, disaggregated stroke data are not yet available [23].

### GLP-1 RAs and cognitive impairment

We found 7 articles related to GLP-1 RAs and cognitive impairment (see Table 2) [24–30], 5 of which were double blind, randomized, placebo-controlled trials [24, 25, 27, 28, 30], while that of Zhang et al. [26] was a randomized clinical trial and that of Li et al. [29] a prospective, parallel, open-label study. All 7 studies included middle- or old-age participants. The studies of Cukierman-Yaffe et al. [24], Zhang et al. [26], Li et al. [29] and Vadini et al. [30] included participants with a diagnosis of type 2 diabetes, whereas the studies of Watson et al. [25], Mullins et al. [27] and Gejl et al. [28] excluded these patients. However, only Zhang et al. [26], Mullins et al. [27] and Gejl et al. [28] included participants with mild cognitive impairment; the remaining 4 studies enrolled those with no cognitive outcomes at baseline.

**Table 2:** Glucagon-like peptide-1 receptor agonists and Cognitive Impairment

Study [Ref.] and year of publication	Study design	Patients, N	Type of participants	Intervention	Neuropsychological and other tests	Blood and CSF tests	Neuroimaging	Main findings	Conclusions
Gejl M. et al. [28] (2017)	Double-blind, randomized placebo-controlled, clinical trial.	N = 38	Participants with Alzheimer’s Disease and not diagnosed with type 2 diabetes	Liraglutide sc. (n = 18) vs. placebo (n = 20)	WMS-IV (brief cognitive examination)	NO	Aβ-protein deposition and CBF ([11 C] PIB PET), CMRglu and ([18 F] FDG PET)	-Liraglutide sc. group, preserved CMRglu compared with placebo, which underwent decline. -No differences in aβ deposition and cognition. -No apoE subgroups reported.	Liraglutide sc. did not show differences between groups in general cognition.
Watson K.T. et al. [25] (2018)	Double-blind, randomized, placebo-controlled trial.	N = 43	Participants between 45–70 years old, absence of type 2 diabetes, MMSE score of > 27 and at least 12 years of education.	Liraglutide sc. (n = 25) vs. placebo (n = 16).	BNT BVRT CVLT-II D-KEFS Pegboard RCFT SDFR WAIS-III WASI: Vocabulary Matrix Reasoning	Plasma biomarkers: -Fasting plasma glucose, -Impaired glucose tolerance	MRI	-Glucose tolerance measured at 120 min declined slightly for the active group (p = 0.06). - Higher FPG was associated with decreased connectivity between bilateral hippocampal and anterior medial frontal structures. -Significant improvement in intrinsic connectivity within the DMN in the active group relative to placebo. -No cognitive differences between study groups.	-Liraglutide sc. showed no cognitive differences between groups (although its design was not focused on detecting these changes).
Mullins R.J. et al. [27] (2019)	Double-blind, randomized placebo-controlled clinical trial.	N = 21	Participants aged > 60 years, absence of diabetes, clinical diagnosis of amnestic Mild Cognitive Impairment or (mild) probable AD.	Exenatide sc. (n = 11) vs. placebo (n = 10).	ADAS-Cog, ADCS-ADL, ANART, Boston Naming Test, BVRT, CDR, CDR-sob, Clock drawing, CVLT, Digit-Symbol and Digit Span Forward & Backward Subtests, FAB, MMSE, Trail-making Test Parts A & B, UPSIT, Wechsler Adult Intelligence Scale, Wechsler Memory Scale Logical Memory Subtest.	CSF biomarkers: -Aβ42 -Total TAU -p181-tau Plasma biomarkers: -Aβ40 -Aβ42 -Fasting glucose -Fasting insulin -OGTT -Homa2 EV biomarkers: -Aβ40 -Aβ42 -Phosphorylated proteins TAU and IRS-1.	MRI MRS	-Better performance for exenatide sc. group in Digit-span forward total score (p = 0.006) and maximum digit-span forward (p = 0.17). -No significant treatment effects on biomarkers in CSF, plasma and EV, except from Aβ42 in EVs, which decreased over time in exenatide-treated group (p = 0.045). -No significant changes were observed over time in MRS metabolites. -No significant effect of exenatide on preserving GM volume or cortical thickness, cortical complexity, gyrification, or sulcal depth.	AD-related outcomes with exenatide sc. cannot demonstrate good findings with the possible exception of a decrease in Aβ42 in neuronal-origin enriched EVs and improvement in attention and memory.
Zhang Z. et al. [26] (2019)	Randomized clinical trial	N = 105	Participants aged between 35 and 70 years old divided in 3 groups: obese + type 2 diabetes, non-obese + type 2 diabetes, and normal control subjects, all three groups with > 6 years of education.	Obese + type 2 diabetes: Liraglutide sc. (n = 10) vs. exenatide sc. (n = 10). Non obese + type 2 diabetes: daily metformin (n = 35) Control subjects: (n = 35).	Cognitive tests 16-word Philadelphia Verbal Learning Test and Wechsler Memory Scale, Animal Naming Test, Boston Naming Test, Digit Span Test, MMSE, MoCA, Stroop Colour and Word Test, Trail Making Test Part A and Part B. Olfactory test OLFACT	Plasma biomarkers: Type 2 diabetes patients: -100 g standard meal test Control patients: -OGTT Both groups: -Glucose levels -Insulin levels -C-peptide -HOMA2	MRI	Obese vs. non-obese type 2 diabetes: -Olfactory threshold score was lower in obese patients (P = 0.028). -Decreased functional connectivity between seed regions and right insula in obese patients (voxel level P < 0.001 and cluster level P < 0.05). -No differences in olfactory identification or memory test scores. Type 2 diabetes vs. control patients: -Decreased activation in left hippocampus (voxel level P < 0.001 and cluster level P < 0.05) more pronounced in obese patients than in non-obese ones. Obese + type 2 diabetes after intervention: -General cognition (MoCA) (p = 0.014) and olfactory function (p = 0.008) were increased after treatment. In particular, the cognitive subdomains of recall memory (p = 0.005) and olfactory identification ability (p = 0.002) were improved. -Significant increase of right parahippocampus activation was observed in obese patients with diabetes compared with baseline (voxel level p < 0.001 and cluster level p < 0.05). -No differences were found after treatment in other cognitive subdomains.	Obese subjects with type 2 diabetes showed bigger impaired cognition and dysfunctional olfaction than the other groups. GLP-1 RAs ameliorated cognitive and olfactory abnormalities in obese subjects with diabetes.
Vadini F. et al. [30] (2020)	Randomized controlled clinical trial.	N = 40	Participants with diagnosis of type 2 diabetes or prediabetes, as well as obesity.	Liraglutide sc. (n = 20) vs. lifestyle counselling (n = 20).	Cognitive assessment of memory, attention and executive control	Biochemical and anthropometric measurements	NO	-Significant increase in short term memory (p = 0.024) and memory composite z-score (p = 0.0065) was observed in the liraglutide sc. exposed subjects.	Liraglutide sc. might slow memory function decline in diabetic patients in early, and possibly preclinical stages of the disease.
Cukierman-Yaffe T. et al. [24] (2020)	Double blind, randomized, placebo-controlled trial.	N = 9,901	Participants aged ≥ 50, newly diagnosed with type 2 diabetes, additional cardiovascular risk factors, glycated haemoglobin of up to 9·5%.	Dulaglutide sc. (n = 4,949) vs. placebo (n = 4,952) once a week.	DSST MoCA	NO	NO	-The reduction in cognitive outcome was non-significant between both treatment groups (p = 0·11). -After post-hoc adjustment for individual standardized baseline scores, the hazard of substantive cognitive impairment was significantly reduced by 14% in those assigned dulaglutide (p = 0·0018).	It is not clear whether once weekly dulaglutide sc. might reduce cognitive impairment in people with type 2 diabetes or not.
Li Q et al. [29] (2021)	Prospective, parallel assignment, open-label, phase III study.	N = 50	Participants aged 18 to 65 years with diagnosis of type 2 diabetes mellitus.	Liraglutide sc. (n = 25) vs. control (oral non-GLP-1 RAs antidiabetic drugs alone or combined with insulin) (n = 25).	Cognitive assessments: -General cognition evaluation (MMSE). -Cognitive subdomains evaluation -Executive function (Memory and executive screening)	Biochemical and anthropometric measurements	fNIRS	-Better scores in all cognitive tests and bigger improvement in memory and attention in liraglutide sc. group (p = 0.04). -Significantly increased activations of the dorsolateral prefrontal cortex and orbitofrontal cortex brain regions were observed in liraglutide group (p = 0.0038). -After liraglutide sc. treatment, cognitive scores were significantly correlated with changes in these activating brain regions (p < 0.05), but no correlation was observed between the changes in cognitive function and changes of body mass index, blood pressure, or glycaemic levels.	Liraglutide sc. improves cognitive decline in patients with type 2 diabetes mellitus. This beneficial effect is independent of its hypoglycaemic effect and weight loss. The optimal intervention should be targeted to cognitive decline in the early stages of dementia.

(11)C-PIB: (11) Carbono-labeled Pittsburgh Compound-B. [18 F] FDG: 18 F-fluorodeoxyglucose. AD: Alzheimer’s Disease. ADAS-Cog: Alzheimer’s Disease Assessment Scale-Cognitive. ADCS-ADL: Alzheimer’s Disease Cooperative Study-Activities of Daily Living Scale. ANART: American National Adult Reading Test. Aβ-protein: Amyloid beta-protein. BNT: Boston Naming Test. BP: Blood Pressure. BVRT: Benton Visual Retention Test. CBF: Cerebral Blood Flow. CDR-sob: Clinical Dementia Rating Sum of Boxes. CDR: Clinical Dementia Rating. CMRglu: Cerebral Metabolic Rate for Glucose Utilization. CSF: Cerebrospinal Fluid. CV: Cardiovascular. CVLT: Californian Verbal Learning Test. D-KEFS: Delis-Kaplan Executive Function System. DMN: Default Mode Network. DSST: Digit Symbol Substitution Test. EV: Extracellular Vesicles. FAB: Frontal Assessment Battery. fNIRs: Functional near-infrared spectroscopy. FPG: Fasting Plasma Glucose. GLP-1 RAs: Glucagon-like Peptide-1 Receptor Agonists. GM: Grey Matter. HBA1C: Haemoglobin A1c. HOMA2: Homeostasis Model Assessment-2 calculator. IRS-1: Insulin Receptor Substrate-1. MACE: Major Adverse Cardiovascular Events. MMSE: Mini-Mental Test. Moca: Montreal Cognitive Assessment. MRI: Magnetic Resonance Imaging. MRS: Magnetic Resonance Spectroscopy. NeuroQOL: Manual for the Quality of Life in Neurological Disorders. OGTT: Oral Glucose Tolerance Test. PET: Positron Emission Tomography. RCFT: Rey Complex Figure Test and Recognition Trial. SC: subcutaneous. SDFR: Short Delay Free Recall. UACR: Urine Albumin-to-Creatinine Ratio. UPSIT: University of Pennsylvania Smell Identification Test. WAIS-III: Wechsler Adult Intelligence Scale-III. WASI: Wechsler Abbreviated Scale of Intelligence. WMS-IV: Wechsler Memory Scale-IV.

All studies measured glucose plasma biomarkers in blood test, except for Cukierman-Yaffe et al. [24] and Gejl et al. [28]. However, Mullins et al. [27] also measured CSF biomarkers as well. All 7 studies used different neuropsychological tests for cognitive assessment. Neuroimaging consisted of MRI acquisition before and after the intervention in all studies, except for the studies of Cukierman-Yaffe et al. [24] and Vadini et al. [30], in which no neuroimaging was performed.

Cukierman-Yaffe et al. [24], using dulaglutide sc. vs. placebo, observed a non-significant reduction in cognitive impairment; p = 0.11. However, after post-hoc adjustments for individual standardized baseline scores, they found a significant reduction in the number of cognitive outcomes; p = 0.0018. In this sense, Zhang et al. [26], included type 2 diabetes mellitus obese patients under GLP1RA treatment vs. type 2 diabetes mellitus non-obese patients under metformin vs. control subjects, and found an increase in general cognition (p = 0.014) and olfactory function (p = 0.008) compared to baseline, in obese participants with type 2 diabetes, particularly, in the cognitive subdomains of recall memory (*p* = 0.005) and olfactory identification ability (*p =* 0.002). Also, a statistically significant increase in right parahippocampus activation was observed in these obese participants, with no differences in other cognitive subdomains. Following these results, Li et al. [28] and Vadini et al. [30], using liraglutide as the intervention, also found an improvement in general cognition. This differs from the findings of Watson et al. [24] and Gejl et al. [28], who used the same intervention but found no cognitive differences between the liraglutide group and placebo. However, Watson et al. [25] did find a significantly better delayed recall and selective attention in comparison to placebo, and a greater positive connectivity between the bilateral hippocampus and preserved amyloid β protein (aβ). On the other hand, Gejl et al. [28] found a preservation of GRM_glu_ compared to placebo, as well as no differences in aβ deposition.

In addition, Mullins et al. [27], using exenatide as the intervention, also failed to observe any differences in cognitive performance between the treatment groups. No changes were observed in MRS metabolites, the effect of exenatide on preserving GM volume or cortical thickness, glucose plasma or CSF biomarkers, except for Aβ_42_ which decreased over time.

### GLP-1 RAs and peripheral neuropathy

We found 4 articles related to GLP-1 RAs and peripheral neuropathy (see Table 3) [31–34]. Those of Wegeberg et al. [31] and Brock et al. [32] were double-blind, randomized, placebo-controlled trials, while the other 2 (Ponirakis et al. [33] and Jaiswal et al. [34]) were open-label, randomized, controlled trials. The studies of Wegeberg et al. [31] and Ponirakis et al. [33] were post-hoc analyses and substudies of the TODINELI and Qatar Study trials, respectively. All four studies enrolled participants aged > 18 years with a diagnosis of diabetes mellitus, but while Wegeberg et al. [31] and Brock et al. [32] included participants with type 1 diabetes, Ponirakis et al. [33] and Jaiswal et al. [34] enrolled those with type 2 diabetes.

**Table 3:** Glucagon-like peptide-1 receptor agonists and Peripheral Neuropathy

Study [Ref.]	Study design	Patients, N	Type of participants	Interventions	Complementary tests	Main findings	Conclusions
Jaiswal M. et al. [34] (2015)	Single centre, proof-of-concept-pilot, open-label randomized, controlled trial.	N = 70	Participants aged between 18 and 70 years and diagnosis of type 2 diabetes.	Twice daily exenatide sc. (n = 22) vs. daily insulin glargine (n = 24).	Skin biopsy for reinnervation, Temperature, Peripheral vibration perception, Cardiovascular reflex assessment by deep breath and Valsalva manoeuvre measure. Quality of life measure.	-Glargine group had a marginally higher regeneration rate after capsaicin denervation at 12 months than exenatide sc. group; 4.6 ± 2.9 fibres/mm (p = 0.002) vs. 2.1 ± 3.5 fibres/mm at 12 months, (p = 0.06). - No group differences were observed over 18 months in either the NeuroQOL scores or overall global quality of life scores.	Exenatide sc. treatment had no significant effect on peripheral neuropathy compared with glargine treatment.
Brock C. et al. [32] (2019)	Prospective, double-blind, randomized, placebo-controlled trial.	N = 114	Participants aged > 18 years and diagnosis of type 1 diabetes.	Liraglutide sc. (n = 28) vs. placebo (n = 20).	-Peripheral neuronal assessment: conduction velocities, amplitude, sensation and temperature. -Autonomic neuronal assessment: electrocardiographic recording by Holter. -Central neuronal assessment: electrical stimulation for somatosensory evoked potential.	-Liraglutide sc. reduced interleukin-6 levels by 22.6%; (P = 0.025). - Neuronal function was unaltered at the central, autonomic or peripheral level and no significant reduction in early precortical latency from evoked brain potentials was observed.	The lowering of the systemic level of proinflammatory cytokines by liraglutide sc. may lead to prevention or treatment of the neuroinflammatory component in early stages of diabetic neuropathy.
Ponirakis G. et al. [33] (2020)	Prospective substudy of the open-label, randomized, placebo-controlled Qatar Study trial.	N = 56	Participants aged between 18 and 75 years and diagnosis of type 2 diabetes.	Exenatide sc. + pioglitazone (n = 21) vs. Glargine + Aspart (n = 17) vs. Controls (n = 18).	Diabetic retinopathy assessment: Digital retinal images of both eyes. Diabetic neuropathic assessment: Corneal nerve density and length by scan. Peripheral vibration perception. Sweat chloride concentrations. Questionnaire for neuropathic pain (DN4).	Glargine + Aspart group: - CNBD and CNFL increased by 27.2 branches/mm2 (p = 0.01) and 2.3 mm/mm2 (p < 0.01), respectively. No changes in CNFD. - Vibration perception threshold decreased by 2.8 V (p < 0.01). - No change in sudomotor function. Exenatide sc. + Pioglitazone group: -CNBD increased by 19.0 branches/mm2 (p = 0.02) with no change in CNFD (p = 0.76) and CNFL (p = 0.12). - Vibration perception threshold increased by 1.7 V (p < 0.05). - No change in sudomotor function.	Treatment with exenatide sc. and pioglitazone or basal-bolus insulin results in corneal nerve regeneration.
Wegeberg A.L. et al. [31] (2020)	Post-hoc analysis of the prospective, randomised, double-blind, parallel-group, placebo-controlled TODINELI trial.	N = 48	Participants aged > 18 years and diagnosis of type 1 diabetes.	Liraglutide sc. (n = 19) vs. placebo (n = 20).	-Gastrointestinal transit time by changes in the pH. -Motility index, by contraction frequency and amplitude.	-Liraglutide sc. treatment reduced large bowel transit time by 31.7%, (p = 0.04) and decreased motility index by 6.1%, (p = 0.04) compared to placebo. -Liraglutide sc. Increased small bowel transit time, which was associated with decreased bloating (p = 0.008). -No differences in gastric emptying or small-bowel transit times between groups were observed.	Liraglutide sc. potentially improves the function of the enteric nervous system by improving the coordination of propulsive motility (accelerating large bowel transit and decreasing motility index).

CNBD: Corneal Nerve Branch Density. CNFD: Corneal Nerve Fibre Density. CNFL: Corneal Nerve Fibre Length. CV: Cardiovascular. DN4: Douleur Neuropathique 4 Questions. GLP-1 RAs: Glucagon-like Peptide-1 Receptor Agonists. HBA1C: Haemoglobin A1c. NeuroQOL: Manual for the Quality of Life in Neurological Disorders. PN: Peripheral Neuropathy. SC: subcutaneous

Neuronal function in patients with type 1 diabetes mellitus was assessed by measuring pH changes, and contraction frequency and amplitude of the gastrointestinal system in the study of Wegener et al. [31], and conduction velocities, amplitude, sensation, temperature, electrocardiographic recording by Holter and electrical stimulation for somatosensory evoked potential in the study of Brock et al. [32] for central, peripheral and autonomic neuronal assessment. However, in type 2 diabetes mellitus patients, the neuronal assessment included peripheral vibration perception and temperature in both studies, but Ponirakis et al. [33] also evaluated corneal nerve density and length by scan and sweat chloride concentrations and used the questionnaire for neuropathic pain (DN4). Jaiswal et al. [34] also performed a skin biopsy to evaluate reinnervation, assessed cardiovascular reflex by the deep breathing test and the Valsalva manoeuvre and asked about quality of life using the Neuropathy Specific Quality of Life Measure.

GLP-1 RAs seem not to have a significant effect on peripheral neuropathy, compared to insulin-treated patients. However, among those participants with type 2 diabetes mellitus receiving exenatide treatment, a significant increase in corneal nerve branch density (p = 0.02) and vibration perception threshold (p < 0.05) were observed by Ponirakis et al. [33] and Jaiswal et al. [34], respectively.

On the other hand, patients with type 1 diabetes mellitus treated with liraglutide, despite showing no significant effects on peripheral neuropathy, did experience an improvement in the function of the enteric nervous system in the study of Wegeberg et al. [31], with a reduction in large bowel transit time (p = 0.04) and motility index (p = 0.04). Brock et al. [32] suggest that the lowering of the systematic levels of proinflammatory cytokines observed, in particular interleukin-6 levels (p = 0.025), may lead to prevention of the neuroinflammatory component in early stages of diabetes neuropathy.

## Discussion

Diabetes is known to be an independent risk factor for cerebrovascular disease. Furthermore, among the causes of stroke, up to 16% have been attributed to diabetes according to some studies [35]. Among people with diabetes, women are at higher risk than men [36] and ischaemic stroke is more frequent than haemorrhagic stroke, probably due to the higher prevalence of hypertension and microvascular disease [37, 38]. In the aetiopathogenesis of stroke, arteriosclerosis is not the only mechanism involved in cerebrovascular disease in patients with diabetes, since there is a greater risk of atrial fibrillation in persons with diabetes, favouring cardioembolic stroke [39]. Diabetes is also associated with increased stroke recurrence, increased disability and mortality [40], and increased risk of developing post-stroke dementia [41].

Some studies have shown that the presence of hyperglycaemia during acute stroke is associated with increased morbidity and mortality regardless of the presence of diabetes. However, intensive treatment with intravenous insulin therapy has not been shown to reduce the prognosis [42]. On the other hand, tight long-term control of HbA1c reduces the risk of microvascular complications in type 2 diabetes [43]. However, it is not entirely clear that HbA1c control by intensive hypoglycaemic therapy reduces the risk of macrovascular complications, including stroke [44, 45].

Since the reduction of glucose concentrations with the classic treatments in diabetes has not given the expected results in terms of cardiovascular protection, the therapeutic strategy with the new antidiabetic drugs such GLP-1 RAs should now focus on the modification of vascular risk factors [3]. Additionally, glucose lowering itself does not seem to be involved in the neuroprotective effect of GLP-1 RAs, thus suggesting that these drugs may be beneficial in stroke in patients both with and without diabetes.

Among the classic drugs for diabetes, two showed beneficial effects in relation to the risk of stroke. In the UKPDS study [46], metformin in type 2 diabetes and overweight was shown to reduce the risk of ischaemic stroke compared to sulfonylureas or insulin. On the other hand, pioglitazone in the PROactive study [47], in patients with type 2 diabetes with symptomatic vascular disease, did not demonstrate a reduction in the risk of stroke, but in a post-analysis in patients with previous stroke it demonstrated a significant reduction in recurrence. Furthermore, a recent meta-analysis proposed pioglitazone as a possibly suitable drug for the secondary prevention of stroke in patients with insulin resistance, prediabetes, and type 2 diabetes [48]. There are no other specific studies with other drugs for diabetes that have shown benefit in the secondary prevention of stroke.

GLP-1 RAs have been shown to cross the blood-brain barrier and their neuroprotective effect seems to be mediated by anti-inflammatory, antioxidant and antiapoptotic effects [49–52]. In preclinical studies models of acute ischaemic stroke have demonstrated a reduction in the volume of the cerebral infarct and better functionality after ischaemic stroke [51, 52]. Additionally, they have been shown to moderately reduce systolic blood pressure and blood lipid concentrations, among other effects [53].

It is important to highlight that there are no clinical trials specifically designed to evaluate the effect of GLP-1 Ras in reducing stroke as the primary objective, so the information is based on data on the risk of stroke as a secondary variable in clinical trials or post-hoc analysis thereof.

The primary objective of most of the large, double-blind, randomized, placebo-controlled trials that have evaluated the cardiovascular effects of GLP-1 RAs in patients with type 2 diabetes focused on evaluating the presence of a MACE. The various subgroup analyses relating to the risk of stroke showed that significant reductions could be achieved with semaglutide sc. by 39% and dulaglutide sc. by 24%. The pathophysiological mechanisms that lead to this reduction in stroke risk are unknown, being attributed to a probable antithrombotic effect over the control of other CVRFs such as blood pressure reduction, lipid control and weight loss.

In this sense, a post-hoc analysis suggested that the reduction in MACE using liraglutide is independent of the previous NYHA functional class [54] or blood pressure levels [55] and this reduction occurs in both patients with or without microvascular disease [56]. A smaller reduction in MACE was also shown in the composite outcome that included expanded MACE rather than MACE (13.4% vs. 15.7%) [57]. Another post-hoc analysis suggested that the reduction in MACE with liraglutide could be mediated by the lowering of HbA_1c_ [58]. Post-hoc analysis of SUSTAIN-6 (semaglutide sc.) showed the same results as those obtained in post-hoc analysis of LEADER (liraglutide sc.) in terms of MACE reduction, despite the presence or otherwise of previous microvascular disease (although this enhances the chances of a MACE) [56] and blood pressure levels [55]. Other post-hoc analyses showed that long-term dulaglutide sc. does not modify stroke severity [59], but they also demonstrated a greater reduction with this drug for the composite outcome that included expanded MACE [60].

On the other hand, it must be taken into account that no clinical trials have been carried out on the secondary prevention of stroke with GLP-1-RAs and most of the data provided are in primary prevention. However, post-hoc analysis of the LEADER and SUSTAIN- 6 have evaluated the effect of liraglutide and semaglutide sc. on the subgroup of patients with previous non-fatal stroke or myocardial infarction [55, 61] and no significant benefit was found in reducing non-fatal stroke, except for liraglutide in patients with glomerular filtration rate < 60 mL/min/1.73 m2 [62]. To date, there are no secondary analyses of the effect of lixisenatide, albiglutide, exenatide, oral semaglutide or dulaglutide in the prevention of recurrences of stroke. Additionally, the LEADER study reported a post-hoc analysis in patients with polyvascular disease (defined as atherosclerosis in two or more of the following vasculature territories: ocular, coronary arteries, cerebral arteries or arteries peripheral), in which they did not observe significant differences in the risk of non-fatal stroke in the liraglutide group versus placebo [63].

Following positive results in preclinical models, GLP1 RAs have been targeted in clinical trials to assess effects on cognition in both type 2 diabetes and Alzheimer’s disease patients [13]. They also have the advantage of not affecting blood glucose levels in non-diabetic people and therefore may represent a possible safe treatment for Alzheimer’s disease in patients without diabetes as they can pass the blood brain barrier. Evidence suggests that GLP-1 RAs are neuroprotective and neurotrophic by providing protection against glutamate-induced apoptotic neuronal cell death and inducing differentiation and neurite outgrowth [14]. Of the literature reviewed here, Zhang et al. [26], Li et al. [28] and Vadini et al. [30] suggested that liraglutide might improve impaired cognition, but this improvement seems to appear only in patients with a diagnosis of type 2 diabetes mellitus, because two studies, Watson et al. [25] and Gejl et al. [28] which involved patients without type 2 diabetes mellitus, did not find significant differences between treatment groups despite some beneficious changes in structures and cognitive subdomains. Nevertheless, these two latter studies leave the door open for future larger and stronger studies to demonstrate significant changes in cognition. In addition, the study of Watson et al. [25] was not designed to detect changes in general cognition. It is also important to mention that the results of the studies by Zhang et al. [26], Li et al. [29] and Vadini et al. [30] may be influenced by the fact that patients with type 2 diabetes who received liraglutide were also patients with obesity and that the control group in the study of Li et al. [29] had a lower BMI than the intervention group. On the other hand, the results with exenatide are controversial, since Mullins et al. [27] found no differences between the treatment groups whereas Zhang et al. [26] did. This situation may be due to the fact that Mullins et al. [27] only included non-diabetic patients in their study whereas Zhang et al. [26] included patients with type 2 diabetes. In addition, exenatide proved to significantly reduce Aβ_42_ in neuronal-origin enriched extracellular vesicles in the study of Mullins et al. [27].

In a post-hoc analysis of the REWIND trial, involving 9901 patients, Cukierman-Yaffe et al. [24] showed that dulaglutide sc. only improved cognitive impairment significantly after adjustments for individual baseline scores, making it clear that more evidence is needed before reaching a conclusion. Of note is the number of patients involved in this post-hoc analysis, unlike the small populations analysed in the other studies of this neurological field.

Diabetic peripheral neuropathy can affect up to 50% of patients with diabetes. While intensive glycaemic control can prevent the onset or delay progression of diabetic peripheral neuropathy in type 1 diabetes, data in type 2 diabetes are conflicting. Both GLP-1 RAs and thiazolidinediones produce a durable reduction in HbA1c [33].

Studies that analyze the effect of GLP-1 analogues in diabetic neuropathy are scarce. However, it seems that neither liraglutide nor exenatide improve the peripheral nerve system in patients with diabetes mellitus. Liraglutide was used in patients with type 1 diabetes, whereas exenatide was use in patients with type 2 diabetes. The former showed an improvement in the enteric nerve system [31] and also lowered the systemic levels of proinflammatory cytokines, which may lead to the prevention of future peripheral neuropathy in early stages of diabetes mellitus, although more evidence is required [32]. On the other hand, exenatide had no significant effect on peripheral neuropathy [33, 34].

This systematic review has potential limitations. It was difficult to extract firm conclusions because of the great heterogeneity of the studies in cognitive impairment and peripheral neuropathy. The sample size, duration and design of the interventions, outcome measures investigated, and analyses performed differed greatly between the studies. Of the studies addressed, 8 had fewer than 100 patients in total. Additionally, some of the studies were of short treatment duration. These facts have an important impact on the interpretation of the results.

## Conclusions

Although there exist strong studies about the effects of GLP-1 RAs on stroke and MACE, more information is required to extrapolate these results to other neurological fields as they seem to be very poor and insufficient in the fields of cognitive impairment and peripheral neuropathy. Nevertheless, dulaglutide in type 2 diabetes mellitus proved to be efficient in decreasing the number of nonfatal strokes. It also seems to have a positive effect on the reduction of cognitive impairment. However, further studies are needed to confirm this latter observation, as this result comes after an adjustment for individual standardized baseline scores. Semaglutide sc. has been shown to reduce nonfatal stroke cases and MACE (when it is administrated subcutaneous and not oral).

Exenatide proved to be effective in improving general cognition and olfactory function when given to patients living with obesity and type 2 diabetes. In patients with type 2 diabetes, it also enhances vibration perception threshold but does not seem to have any effect on reducing stroke cases, though it does appear to reduce MACE. Liraglutide improved general cognition and olfactory function only when given to patients living with obesity and type 2 diabetes. It reduces large bowel transit time in type 1 diabetes mellitus, improving the motility index, but increasing the small bowel transit time as well. Although liraglutide reduces the number of MACE in patients with type 2 diabetes, it does not have the same effect on stroke. Lixisenatide, albiglutide or efpeglenatide in type 2 diabetes mellitus have not been shown to reduce the number of fatal or nonfatal stroke cases, but the latter two reduce the number of MACE.

More studies are required in order to extend our knowledge and understand the effects of GLP1-RAs on neurological complications of diabetes.

## Abbreviations

CVRF: Cardiovascular risk factor.
GLP-1: RAs Glucagon-like peptide-1 receptor agonists.
MACE: Cardiovascular safety profile of currently available diabetic drugs.
